# Case report: Second report of neuromuscular syndrome caused by biallelic variants in *ASCC3*

**DOI:** 10.3389/fgene.2024.1382275

**Published:** 2024-09-02

**Authors:** Wang Li, Zhongliang Li, Junhui Fu, Kaili Xu, Daoqi Mei, Xiaona Wang, Taisong Li, Xilong Du

**Affiliations:** ^1^ Department of Neurology, Children’s Hospital Affiliated of Zhengzhou University, Zhengzhou, China; ^2^ Department of Neurology, Henan Children’s Hospital, Zhengzhou, China; ^3^ Department of Neurology, Zhengzhou Children’s Hospital, Zhengzhou, China; ^4^ Department of Neonatology, Weifang Maternity and Child Care Hospital, Weifang, China; ^5^ Department of Rehabilitation Medicine, Zhoukou Sixth People’s Hospital, Zhoukou, China; ^6^ Henan Children’s Neurodevelopment Engineering Research Center, Children’s Hospita Affiliated to Zhengzhou University, Zhengzhou, China; ^7^ Henan Key Laboratory of Children’s Genetics and Metabolic Diseases, Children’s Hospital Affiliated of Zhengzhou University, Zhengzhou, China; ^8^ Beijing Chigene Translational Medical Research Center, Beijing, China

**Keywords:** *ASCC3*, developmental delay, intellectual disability, whole-exome sequencing, neuromuscular syndrome

## Abstract

**Introduction:**

Activating Signal Cointegrator 1 Complex, Subunit 3 (*ASCC3*) has been implicated in the pathogenesis of neurodevelopmental disorders and neuromuscular diseases (MIM: 620700). This paper analyzes the clinical manifestations of three patients with developmental delay caused by *ASCC3* genetic variation. Additionally, we discuss the previously reported clinical features of these patients along with our own findings, thereby enhancing our understanding of these genetic disorders and providing valuable insights into diagnosis, treatment, and potential interventions for affected individuals.

**Methods:**

In this study, we utilized trio-whole-exome sequencing (Trio-WES) and trio-copy number variations sequencing (Trio-CNV-seq) to analyze three unique families diagnosed with developmental delay caused by variation in *ASCC3*. Additionally, we retrospectively examined eleven previously reported *ASCC3* genetic variations exhibiting similar clinical features.

**Results:**

Proband I (family 1) and Proband III (family 3) exhibited global developmental delays, characterized by intellectual disability, motor impairment, language retardation, lower muscle strength, and reduced muscle tone in their extremities. Proband II (family 2) presented poor response and dysphagia during feeding within 7 days after birth, clinical examination displayed short limbs, long trunk proportions, and clenched fists frequently observed alongside high muscle tone in his limbs -all indicative signs of developmental delay. Trio-WES revealed compound heterozygous variants in *ASCC3* inherited from their parents. Proband I carried c. [489 dup]; [1897C>T], proband II carried c. [2314C>T]; [5002T>A], and proband III carried c. [5113G>T]; [718delG] variations, respectively.

**Conclusion:**

This study present the first report of Chinese children carrying compound heterozygous genetic variants in *ASCC3* with LOF variants, elucidating the relationship between these variants and various aspects of intellectual disability. This novel finding expands the existing spectrum of *ASCC3* variations.

## 1 Introduction

Global developmental delays (GDD) encompass a multifaceted condition characterized by heterogeneous manifestations, influenced by diverse genetic and environmental factors. Genetic factors encompass chromosomal abnormalities, monogenic diseases, mitochondrial disorders, as well as polygenic and/or epigenetic abnormalities. A comprehensive search of the online Mendelian Inheritance in Man (OMIM) and National Center for Biotechnology Information (NCBI) databases was conducted using terms such as “global developmental delay, intellectual disability,” resulting in an increase in the number of associated pathogenic genes from 818 in 2014 to 920 presently (Vissers, Gilissen, and Veltman, 2016; Chiurazzi and Pirozzi, 2016). Given the broad clinical phenotype and genetic heterogeneity observed in GDD, distinguishing phenotypes and genetic patterns can be challenging, potentially leading to diagnostic delays. However, with the integration of genetic testing technologies into clinical practice, there has been significant improvement in diagnosing genetic etiologies.

*ASCC3* (Activating Signal Cointegrator 1 Complex, Subunit 3) was initially identified as a gene associated with intellectual disability (ID) and cognitive impairment by Najmabadi H (MIM: 620700) (Najmabadi et al., 2011). They found that homozygous variants in ASCC3 were linked to intellectual disability in four individuals from one family, and the phenotype segregated separately from unaffected members. Through a genome-wide association study (GWAS), Chen CH et al., discovered 11 new loci involved in various neuro-related phenotypes, including *ASCC3* (Chen et al., 2017). Recently, biallelic variants in *ASCC3* have been reported in association with intellectual developmental disorder. The authors reported *ASCC3* as a causative gene for a neuromuscular syndrome, in seven patients from six families (Nair et al., 2021).

This report presents three Chinese children with *ASCC3* gene variants associated with neuromuscular disorder.

## 2 Materials and methods

### 2.1 Sample collection

The patient’s guardian and the Institutional Review Board of our hospital (IRB:2024-K-059) provided informed consent. Whole-exome sequencing and copy number variation sequencing were conducted on both the patient and their family members. The patients were enrolled in accordance with the outlined sequence described in the article.

### 2.2 DNA extraction

The umbilical cord blood or fetal tissue genomic DNA was extracted using the Blood Genome Column Medium Extraction Kit (Kangweishiji, China) in accordance with the manufacturer’s instructions. The extracted DNA samples were subjected to quality controlling using Qubit 2.0 fluorimeter and electrophoresis with 1% agarose gel for further processing.

### 2.3 Whole exome library construction

Protein-coding exome enrichment was conducted using xGen Exome Research Panel v2.0(IDT, Iowa, United States), which consists of 429,826 individually synthesized and quality-controlled probes, and targets 39 Mb protein-coding region (19,396 genes) of the human genome and covers 51 Mb of end-to-end tiled probe space.

### 2.4 Sequencing

The MGISEQ-T7 series sequencer was utilized for high-throughput sequencing, ensuring that no less than 99% of the target sequence was successfully sequenced. The sequencing process was conducted by Beijing Chigene Translational Medicine Research Center Co., Ltd, 100875, Beijing.

### 2.5 SNP and short indel analysis

The paired-end reads were aligned to the Ensembl GRCh37/hg19 human reference genome using the Burrows-Wheeler Aligner (BWA) software (Li and Durbin,2010). Subsequently, single nucleotide variants (SNVs) and small insertions/deletions (InDels) were called using the Genomic Analysis Toolkit (GATK) software (version 4.1.7) (McKenna et al., 2010). The genetic relationship was determined by KING (Manichaikul et al., 2010), and the coverage of the SRY gene was calculated through gender quality control. The identified variants were then annotated utilizing an online system developed by Chigene (www.chigene.org), which incorporates a comprehensive set of 35 public databases. For minor allele frequency annotation, databases such as 1,000 genomes, dbSNP, ESP, ExAC, and Chigene’s in-house MAFs database were employed. To predict structural variations in protein products caused by these variants, Provean, Sift, Polypen2_hdiv, Polypen2_hvar, Mutationtaster, M-Cap, and Revel software packages were utilized. Additionally, the functional impact on splicing sites was predicted using MaxEntScan, dbscSNV, and SpliceAI software packages. Candidate SNVs/small InDels were confirmed by Sanger sequencing and the primer information for the variants reported in this article can be found in Supplementary Table S1.

We classified the candidate variants according to the American College of Medical Genetics and Genomics (Richards et al., 2015) and Sequence Variant Interpretation Working Group international guidelines (SVI WG, https://www.clinicalgenome.org/working-groups/sequence-variant-interpretation/).

### 2.6 CNV analysis

Sequencing reads were aligned to the human reference genome GRCh37/UCSC hg19 using BWA-MEM (Li and Durbin, 2010), Subsequently, SAMtools (Li et al., 2009) and Picard (https://broadinstitute.github.io/picard/) were performed to sorting and marking, respectively. CNVnator (Abyzov et al., 2011) and AMYCNE (Eisfeldt et al., 2018; Geoffroy et al., 2018) software packages were used to detect CNVs. Repeat expansions at known loci were called by Expansion Hunter (Dolzhenko et al., 2019).

SVs and CNVs were annotated and ranked by AnnotSV (Geoffroy et al., 2018), they were evaluated by comparison with literature values and databases, such as the Database of Genomic Variants (DGV, http://dgv.tcag.ca/dgv/app/home), the Database of genomic variation and Phenotype in Humans Using Ensembl Resources (DECIPHER, https://www.deciphergenomics.org/), ClinGen Dosage Sensitivity Map (ClinGen, https://www.clinicalgenome.org/) and PubMed (https://pubmed.ncbi.nlm.nih.gov/).

According to the guidelines of the American College of Medical Genetics and Genomics (ACMG) and the Clinical Genome Resource (ClinGen) (Richards et al., 2015; Brandt et al., 2020; Riggs et al., 2020), the clinical significance of all identified variations was interpreted and classified into five categories: pathogenic (P), likely pathogenic (LP), variant of uncertain significance (VUS), likely benign, and benign.

### 2.7 Clinical phenotypes analysis

The clinical phenotypes of patients were extracted to specific Human Phenotype Ontology (HPO, https://hpo.jax.org/) terms to facilitate identifying disease-causing variants.

### 2.8 Filtering strategy

The filter process was based on a combination of biologic pathogenicity, including the ACMG rating and alignment of the patient’s clinical and gene-disease features, as well as genetics such as mode of disease inheritance and familial gene-phenotype cosegregation. In general, the phenotype and disease characteristics exhibit a high degree of congruence in cases where a genetic disorder is classified by ACMG as likely pathogenic or a pathogenic variant, and the family’s gene-phenotype cosegregation must be satisfied before considering the variant as the cause of disease in the patient.

### 2.9 Computational modelling

The structure of Activating signal cointegrator 1 complex subunit 3, the protein encoded by ASCC3, was modelled using AlphaFold (https://alphafold.ebi.ac.uk/entry/Q8N3C0) to predict the effect of missense variants on protein structure. Hydrogen and clashes bonding were analysed and visualized using UCSF ChimeraX software (Meng et al., 2023).

## 3 Retrospective analysis

We collected variant information and clinical features of 11 previously reported patients for analysis (Najmabadi et al., 2011; Nair et al., 2021; Johari M et al., 2022).

## 4 Results

### 4.1 Case presentation

Proband I (family 1): The patient is a 5-year and 9-month-old male of Han ethnicity. He presented with “intellectual disability” as the chief complaint, exhibiting limited ability to articulate coherent sentences, only capable to forming short phrases consisting of three or four words. He experiences fatigue following brief periods of physical activity. There were no significant complications after birth, feeding difficulties, or swallowing disorders observed. Independent walking commenced at one and a half years old. No notable family history was reported. Physical examination revealed measurements including height of 110 cm (-1SD ∼ -2SD), weight of 20 kg (>-1SD), head circumference of 50.2 cm (>-1SD), palmar-plantar contact, normal tooth distribution, unremarkable facial features, normal vision without strabismus; mild autistic tendencies, reduced muscle tone, poor fine motor skills; symmetric tendon reflexes, negative pathological signs, stable gait, and no cerebellar ataxia. Additional investigations showed normal blood myocardial enzyme spectrum, normal bone age, normal head MRI, normal electromyography, an IQ score of 52 on intelligence testing.

**FIGURE 1:**
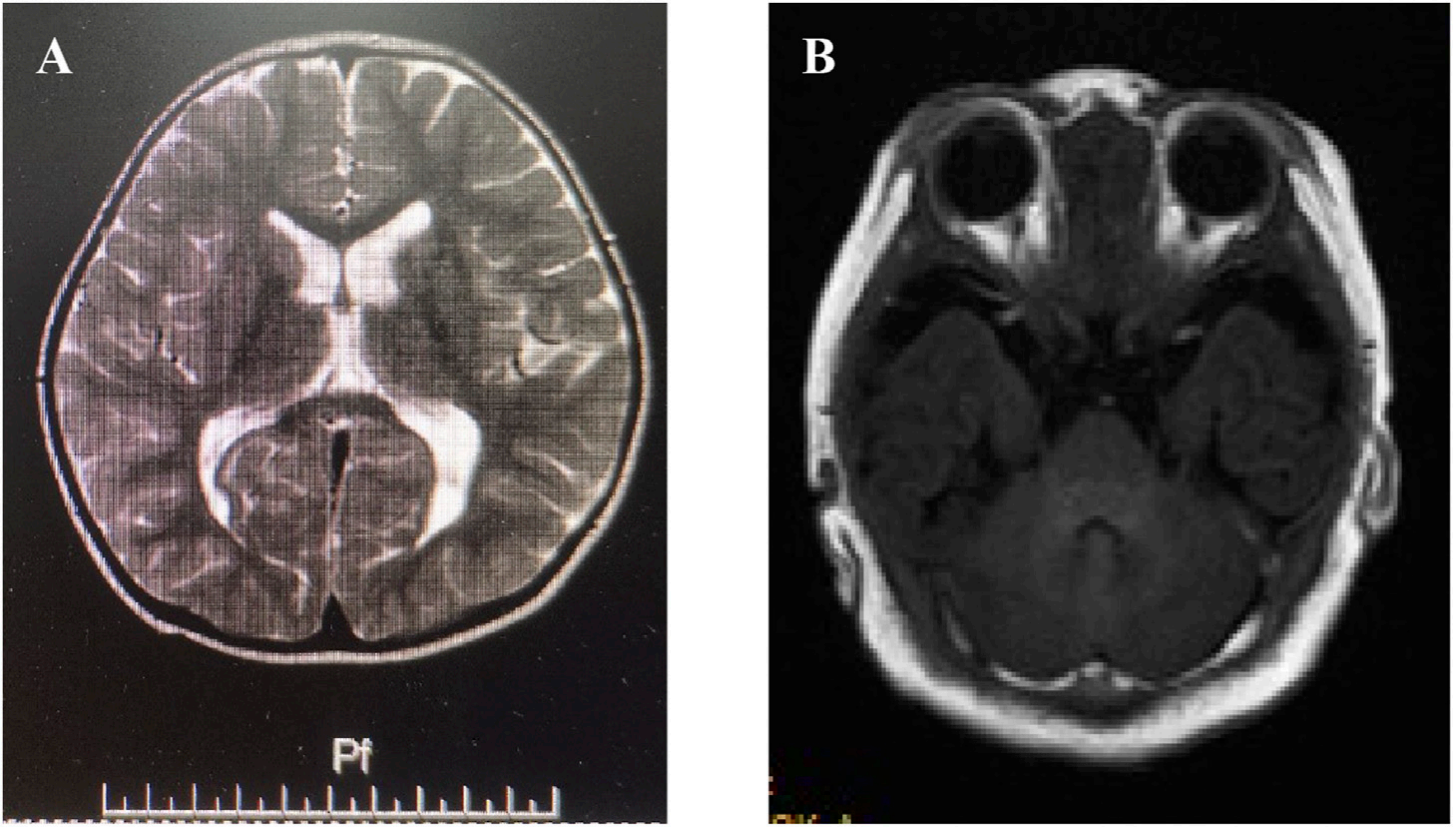
Imaging features of proband II & III. **(A)** Brain magnetic resonance imaging (MRI) displayed small focal signal abnormalities in the bilateral occipital lobes in proband II. **(B)** Brain MRI showed slightly thinner genu and splenium of the corpus callosum along with irregular bilateral lateral ventricles, mildly enlarged posterior horn, and widened left temporal subarachnoid space in proband III.

Proband II (family 2), a male neonate, was admitted to the hospital 7 days after birth due to poor responsiveness and feeding. The infant was delivered at 39^+2^ weeks of gestation without any history of asphyxia and weighed 4490g (+2SD∼3 + SD). The child was irritable, pronounced tremors upon stimulation, accompanied by laryngeal stridor, diffuse cutaneous flushing, bilateral palmoplantar hyperkeratosis, and increased muscle tone in the limbs. Additionally, He presented with short limbs, elongated trunk, macrocephaly, developmental delay, high palatal arch, and generalized eczematous lesions. Urine analysis revealed normal results while methylmalonic acidemia combined with homocysteinemia were detected. Electroencephalogram (EEG) demonstrated moderate abnormal electrical activity, whereas brain magnetic resonance imaging (MRI) displayed small focal signal abnormalities in the bilateral occipital lobes (Figure 1A). Unfortunately, the child succumbed during the recent follow-up, and the parents declined to disclose the underlying cause.

Proband III (family 3), was a female toddler, aged 2 years and 2 months. The patients presented with global developmental delays, speech impairments, cognitive deficiencies, sports developmental delays, delayed motor skills development in sports activities, partial articulation of different words, limited comprehension of instructions, abnormal gait posture characterized by instability and widened foot spacing. No apparent facial abnormalities were observed except for mild flaps and high bowing of the forehead (Hubei sign). The mother had an uneventful pregnancy but underwent a cesarean section due to oligohydramnios. The proband’s head circumference measured 44.5 (<-2SD) cm while exhibiting slightly reduced muscle strength and limb tension. MRI showed slightly thinner genu and splenium of the corpus callosum along with irregular bilateral lateral ventricles, mildly enlarged posterior horn, and widened left temporal subarachnoid space (Figure 1B).

**FIGURE 2:**
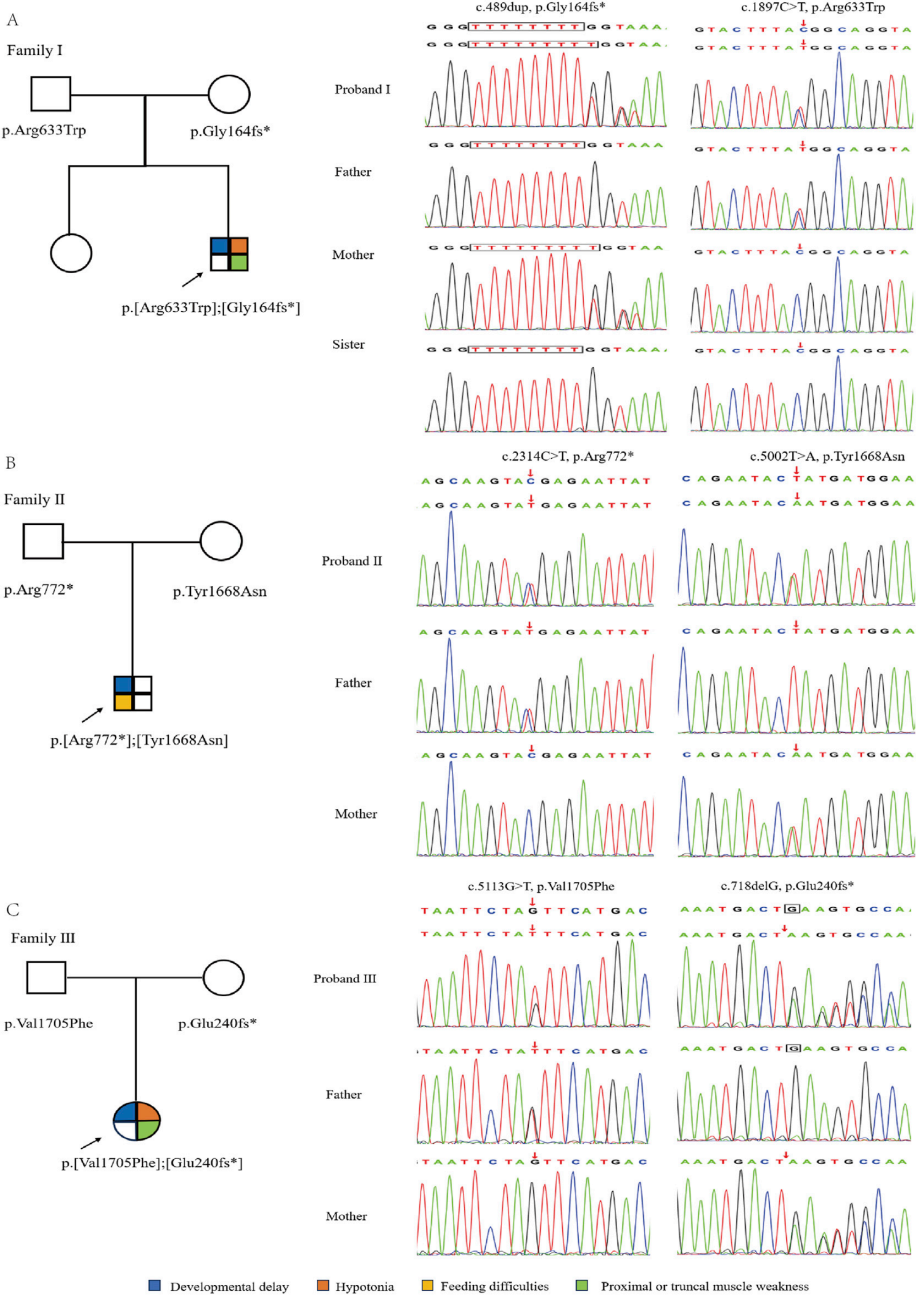
Genetic information of three families. **(A–C)** is the pedigree and sanger sequencing chromatogram of proband I, II & III, respectively.

Whole-exome sequencing of three children with intellectual disability in our hospital identified compound heterozygous variants related *ASCC3* gene. Proband I: c.489dup (p.Gly164fs*) inherited from the mother and c.1897C>T (p.Arg633Trp) inherited from the father; Proband II: c.2314C>T (p.Arg772*) inherited from the father and c.5002T>A (p.Tyr1668Asn) inherited from the mother; Proband III: c.5113G>T (p.Val1705Phe) inherited from the father and c.718delG (p.Glu240fs*) inherited from the mother, respectively (Table 1; Figure 2). The gender and relatedness of the three families were confirmed through bioinformatics analysis of WES data.

**TABLE 1:** Genetic test results and ACMG guideline ratings.

Individual	Proband I (family 1)		Proband II (family 2)		Proband III (family 3)	
Variants	c.1897C>T, p.Arg633Trp (pat)	c.489dup, p.Gly164fs* (mat)	c.2314C>T, p.Arg772* (pat)	c.5002T>A, p.Tyr1668Asn (mat)	c.5113G>T, p.Val1705Phe (pat)	c.718delG, p.Glu240fs* (mat)
CADD Score	31	—	37	26.3	28.5	—
Highest gnomAD Allele Population Frequency	3.99e-6	0	2.84e-05	4.01e-6	0	0
ACMG	VUS: PM2_Supporting + PP3	VUS: PM2_Supporting	VUS: PM2_Supporting	VUS: PM2_Supporting + PP3	VUS: PM2_Supporting + PP3	VUS: PM2_Supporting

According to the guidelines of the American College of Medical Genetics and Genomics (ACMG) in 2015 (Richards et al., 2015), c.1897C>T, c.5002T>A and c.5113G>T were categorized as “uncertain significance” (PM2_Supporting +PP3), while c.489dup, c.2314C>T and c.718delG were classified as “uncertain significance” PM2_Supporting). These findings suggest a certain correlation between variations in *ASCC3* with neuromuscular disorder. In addition, a set of genes associated with intellectual disability was also excluded, and showed the related suspicious variations information (Supplementary Table S2).

**FIGURE 3:**
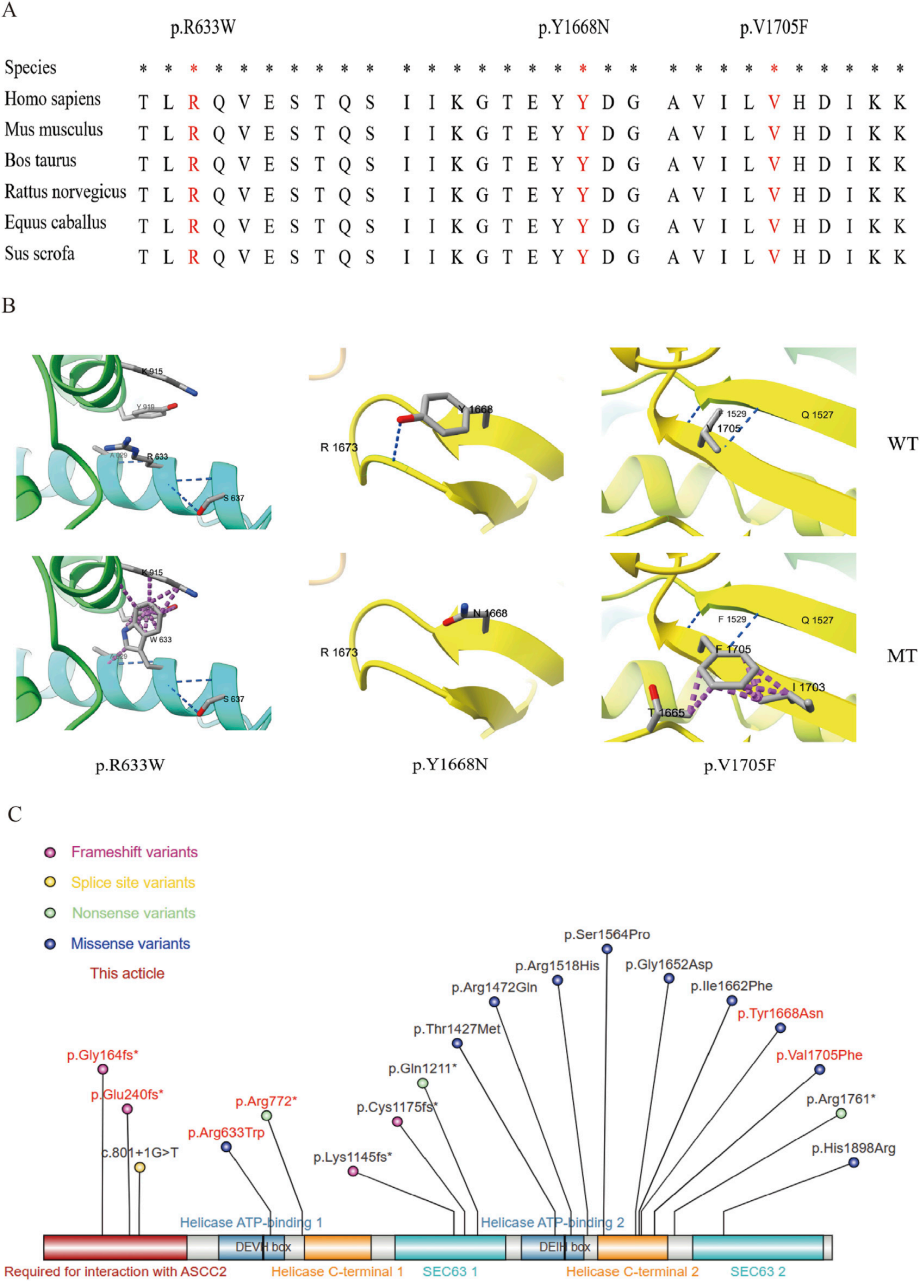
Nucleic acid and amino acid information of the variants. **(A)** Conservation analysis of the three missense variants. *: Highly conserved in multiple species. The red background shows the variant amino acid sequence information. **(B)** Local diagram of the number of hydrogen and clasher bonds changed in ASCC3 homologous protein (NM_006828). Variation: p.Y1668N, the hydrogen bond disappeared before (top) and after (bottom) variation; p. R633W and p.V1705F, although the hydrogen bonds were not changed before and after the variation, the side-chain r group was mutually exclusive with other amino acid atoms (purple), which may affect the correct folding of their active conformation. **(C)** All the variation information of *ASCC3* variations causing neurodevelopmental disorders, and the red background is the variation found in this study.

The three reported missense variations all occurred at highly conserved amino acid positions (Figure 3A). Through surface modeling of the protein tertiary structure, it was observed that the mutant amino acids can potentially disrupt hydrogen bonding with neighboring residues and alter atomic interactions, leading to conformational changes in the spatial structure of the protein. These alterations have the potential to impact the functional characteristics of the protein (Figure 3B). Variate p.Y1668N, the hydrogen bond disappeared before (top) and after (bottom). Variates p. R633W and p.V1705F, although the hydrogen bonds were not changed before and after the variation, the side-chain R group was mutually exclusive with other amino acid atoms (purple). Furthermore, we compiled eighteen *ASCC3* variants associated with autosomal recessive mental developmental disorder 81 (MIM: 62070). Among them, missense mutation (10), frameshift mutations (4), nonsense mutations (3), splice site mutation (1), and highlighted in this paper are those carried by patients indicated with a red background (Figure 3C).

## 5 Discussion

The *ASCC* family (1, 2, 3), also known as the Activating Signal Cointegrator 1 Complex, Subunit 1/2/3, is associated with transcription factors or nuclear receptors, and functions as a bidirectional link between co-repressors and co-activators. It’s playing a crucial role in the regulation of receptor and transcriptional mechanisms, pre-mRNA processing, as well as splicing control. *ASCC3* encodes a dual-cassettes Ski2-like RNA helicase, which unwinds DNA by translocating from the 3′to 5′direction on a single strand. Under the influence of α-ketoglutarate-dependent dioxygenase AlkBH3, an important DNA repair factor, it provides single-stranded DNA to repair alkylation damage (Jia et al., 2020).

ID is a prominent causes of childhood disability globally. In the United States, the prevalence is 1.2% (Maenner et al., 2016), while in Europe, it is less than 1.0%, with severe intellectual disability ranging from 0.3% to 0.4 (Beadle-Brown and Mansell, 2003). There are over 900 genetic disorders associated with ID, where chromosomal abnormalities, including numerical and structural aberrations, account for 25%–30% of all genetic factors. Conventional chromosomal karyotyping analysis can identify genetic etiology in 5%–10% of patients (Flore and Milunsky, 2012). Furthermore, autosomal dominant inheritance contributes to approximately 13%–39% of ID or GDD (Moeschler et al., 2014), with *de novo* variations being a significant cause of severe ID or GDD (de Ligt et al., 2012). Autosomal recessive inheritance accounts for 10%–20% case of ID or GDD (Musante and Hilger Ropers, 2014), particularly prevalent among consanguineous families.

Homozygous or compound heterozygous variants of *ASCC3* have been reported to be associated with neurological disorders and neuromuscular syndromes (Nair et al., 2021; Johari M et al., 2022). All individuals exhibited neurologic phenotypes ranging from mildly developmental delay to muscle fatigue, with prominent features of developmental delay (21/21), hypotonia (11/17), feeding difficulties (4/9), and proximal or truncal muscle weakness (12/17) (Table 2). The clinical symptoms caused by *ASCC3* variations are genetically related. Johari M et al., conference report revealed that in addition to ocular involvement and bulbar weakness, six patients also presented with Congenital myasthenic syndromes (CMS) and other symptoms. Feeding difficulties were mentioned as well; however, the information regarding the published gene variants was not provided.

**TABLE 2:** Synopsis of primary clinical presentations associated with biallelic ASCC3.

	Nair et al. (2021)	Johari M et al. (2022)	This report	Total (n = 21)
Developmental delay	10/10	8/8	3/3	21/21 (100%)
Hypotonia	5/6	4/8	2/3	11/17 (64.7)
Feeding difficulties	3/6	NA	1/3	4/9 (44.4%)
proximal or truncal muscle weakness	2/6	8/8	2/3	12/17 (70.6%)

^a^ NA, not applicable.

Most patients exhibit intellectual developmental delay or motor impairments from birth. Similarly, in our case, patient I and patient III had obvious intellectual and language impairment, movement disorders, and reduced muscle tone. patient I changed from non-gender identification to gender identification 3 months before recovery. Subsequently, global development improved with age during a rehabilitation period lasting more than 1 year; However, he was still significantly behind normal children and showed more pronounced cognitive deficits. Patient Ⅱ had developmental delay since birth, Unfortunately, due to objective reasons patients are unwilling to establish contact with us. In the most recent follow-up, the child passed away and the parents were hesitant to disclose specific causes. Our analysis of whole exome sequencing (WES) data did not reveal any pathogenic variants associated with combined methylmalonic acidemia and homocysteinemia. Additionally, it is worth noting that the patient’s early electroencephalogram displayed abnormalities which do not exclude the possibility of subsequent secondary epilepsy. The clinical phenotypes of all *ASCC3* variations exhibit a high degree of heterogeneity, yet they all manifest with certain intellectual developmental disorders and motor impairments. Reassuringly, no specific facial abnormalities or seizures were observed in our patient cohort, thus justifying their active participation in rehabilitation.

We present three patients exhibiting a more severe phenotype, characterized by compound heterozygous variants in both LOF and missense variant within the *ASCC3* gene. Consistent with Najmabadi H’s hypothesis, biallelic missense variants tend to manifest as a less severe phenotype. Unfortunately, due to patient nonadherence, it was not feasible to further examine alterations in the abundance of ASCC3 protein, transcript levels, or splicing patterns. We assume that these differences may be related to hypomorphic variations. In contrast to loss of function variants, hypomorphic variants do not completely abolish the gene product but cause quantitatively diminished, qualitatively unaltered protein function. It may lead to a milder phenotype in patients with homozygous missense variants (Zschocke et al., 2023). Due to the clinical heterogeneity, we encountered challenges in statistical analysis and were unable to detect any significant differences between males and females. In addition, given the limitations of WES and CNV-seq, although several CNVs of uncertain significance that we detected did not contain *ASCC3*, we cannot exclude that smaller CNVs contain *ASCC3* genes.

In conclusion, this study presents the first report of three patients with *ASCC3* variations associated with developmental delay and muscle fatigue in Chinese children. In conjunction with the analysis of 11 previously reported patients with *ASCC3* variation-related hereditary intellectual developmental disorders, the conclusion is limited due to the limited sample sizes. Therefore, it is imperative to expand the sample size and gather more information on patients with *ASCC3* variation-related hereditary intellectual developmental disorders for further research. Simultaneously, it is crucial to promptly enhance the comprehensive assessment for children with congenital intellectual disability and neuromuscular disorders, taking into account the impact of genetic variations, particularly *ASCC3*.

## Data Availability

The original contributions presented in the study are included in the article/Supplementary Material, further inquiries can be directed to the corresponding authors.
