# Supplementary material for: Case report: Second report of neuromuscular syndrome caused by biallelic variants in ASCC3
Source: Front Genet. 2024 Sep 2;15:1382275. doi: 10.3389/fgene.2024.1382275 (PMC11402803; doi:10.3389/fgene.2024.1382275)
Supplement: Supplementary file 2 [file DataSheet1.PDF]

Case Report: Second Report of Neuromuscular Syndrome Caused by Biallelic Variants in ASCC3

Abstract

Recent research suggests that mutations in *ASCC3* may contribute to the development of neurodevelopmental disorders and neuromuscular diseases. This paper examines the clinical manifestations of three patients with developmental delay caused by *ASCC3* mutation. Additionally, we discuss previously reported clinical features of these patients along with our own findings to enhance our understanding of these genetic disorders and provide insights into diagnosis, treatment, and potential interventions for affected individuals.

Methods: Three patients diagnosed with developmental delay caused by *ASCC3* mutation were selected for this study using whole-exome sequencing. We retrospectively analyzed eleven previously reported *ASCC3* variants and gene mutations with similar clinical features.

Results: Proband I and III exhibited global developmental delays characterized by intellectual disability, motor impairment, language retardation, low muscle strength, and reduced muscle tone in their extremities. Proband II showed poor response and dysphagia during feeding within seven days after birth; clinical examination revealed short limbs, long trunk proportions, and clenched fists frequently observed alongside high muscle tone in his limbs - all indicative signs of developmental delay. Trio-whole-exome sequencing identified compound heterozygous variants in ASCC3 inherited from their parents respectively: c.489_c.490insT and c.1897C>T for proband I; c.2314C>T and c.5002T>A for proband II; c5113G>T and c718delG for proband III. Importantly, our findings further validate that patients harboring loss-of-function (LOF) variants exhibit more pronounced intellectual disability and muscular abnormalities without seizures compared to those with homozygous missense variants.

Conclusion: This study presents the first report of Chinese children carrying a double allelic gene mutation in *ASCC3* with LOF variants

Keywords

ASCC3, developmental delay, intellectual disability, whole-exome sequencing, neuromuscular syndrome

Introduction

It should be noted that GDD exhibits a wide range of manifestations influenced by various genetic and environmental factors. It is important to acknowledge the challenges in distinguishing phenotypes and genetic patterns in GDD due to its extensive clinical phenotype and genetic heterogeneity. However, significant progress

has been made in integrating genetic testing technologies into clinical practice for diagnosing genetic causes and reducing diagnostic delays. Notably, *ASCC3* has been identified as a gene associated with intellectual disability (ID) and cognitive impairment. Previous studies have linked homozygous variants in ASCC3 to ID within one family while segregating separately from unaffected members(Najmabadi et al. 2011). Furthermore, a genome-wide association study discovered 11 new loci involved in neuro-related traits, including *ASCC3*(Chen et al. 2017). Recent findings have also revealed associations between homozygous/compound heterozygous variants in *ASCC3* and neuromuscular syndromes observed in seven individuals from six unrelated families(Nair et al. 2021). An update on a family affected by cognitive impairment/intellectual disability related to mutations in the *ASCC3* gene will be provided. Additionally, three Chinese children diagnosed with conditions related to *ASCC3* will be presented.

Narrative

Proband I, aged 1 year and 9 months, was diagnosed with intellectual disability and post-exercise fatigue. On September 19, 2022, the child received a diagnosis of neurodevelopmental disorder with *ASCC3* gene mutation based on genetic testing data. Proband II presented significant jitteriness after stimulation and high limb muscle tension upon admission at four days old due to poor nursing. The patient exhibited short limbs, a long trunk, and delayed development. Electroencephalography (EEG) revealed moderately abnormal electrical activity, while magnetic resonance imaging (MRI) of the brain showed small focal signal abnormalities in the bilateral occipital lobes. Although the gene test yielded negative results in June 2021, it was re-analyzed in December 2022 leading to a diagnosis of neurodevelopmental disorder syndrome caused by *ASCC3* gene mutation. Proband III, aged two years and two months old, had a clinical diagnosis of comprehensive developmental delays including speech delay, cognitive impairment, and motor developmental delays with slightly reduced lower limb muscle strength and muscle tone. Cranial MRI indicated thinning of the corpus callosum knee region and mild left temporal subarachnoid widening. Genetic testing initially yielded negative results but subsequent analysis in December 2022 confirmed a diagnosis of neurodevelopmental disorder syndrome caused by *ASCC3* gene mutation.

Patient Perspective

All children received appropriate rehabilitation treatment during hospitalization. Unfortunately, only proband I exhibited improved language expression ability following rehabilitation treatment and subsequent hospitalization. Once the pathogenic gene was identified, it posed a challenge as there were no specific treatments available for the disease. Therefore, In proband II and III, despite data reanalysis to identify the causative gene and timely patient follow-up analysis, patients and their families had poor compliance with rehabilitation, and thus limited information was available for children 2 and 3.

Discussion

Homozygous or compound heterozygous variants of *ASCC3* have been reported to be associated with neurological disorders and neuromuscular syndromes (Nair et al. 2021). All individuals exhibited neurologic phenotypes ranging from mild developmental delay to muscle fatigue, with prominent features of developmental delay (10/10) and reduced muscle tone (5/10). The clinical symptoms caused by ASCC3 mutations are genetically related. Most patients exhibit intellectual developmental delay or motor impairments from birth. Similarly, in our case, patient I and patient III had obvious intellectual and language impairment, movement disorders, and reduced muscle tone. Patient II had a developmental delay since birth, Unfortunately, due to objective reasons patients are unwilling to establish contact with us. The clinical phenotypes of all *ASCC3* mutations exhibit a high degree of heterogeneity, yet they all manifest with certain intellectual developmental disorders and motor impairments. Reassuringly, no specific facial abnormalities or seizures were observed in our patient cohort, thus justifying their active participation in rehabilitation.

With the development of detection technology, more and more pathogenic genes of genetic diseases have been found, and the re-analysis of genetic testing data is necessary. This provides value not only for clinical diagnosis but also for future treatment.

Conclusion

In conclusion, this is the first report of three children with *ASCC3* variants associated with developmental delay and muscle fatigue. Comprehensive evaluation of children with congenital mental retardation and neuromuscular disorders should be strengthened in time, considering the influence of genetic variants, especially *ASCC3*.

Acknowledgements

We thank the patients and their parents for their participation in this study.

References

Chen, Chi-Hua, Yunpeng Wang, Min-Tzu Lo, Andrew Schork, Chun-Chieh Fan, Dominic Holland, Karolina Kauppi, Olav B. Smeland, Srdjan Djurovic, Nilotpal Sanyal, Derrek P. Hibar, Paul M. Thompson, Wesley K. Thompson, Ole A. Andreassen, and Anders M. Dale. 2017. "Leveraging genome characteristics to improve gene discovery for putamen subcortical brain structure." Scientific Reports 7 (1). doi: 10.1038/s41598-017-15705-x.

Nair, Divya, Dong Li, Hannah Erdogan, Andrew Yoon, Margaret H. Harr, Gaber Bergant, Borut Peterlin, Maruša Škrjanec Pušenjak, Parul Jayakar, Rolph Pfundt, Sandra Jansen, Kirsty McWalter, Alpa Sidhu, Sheila Saliganan, Emanuele Agolini, Arthur Jacob, Jennifer Pasquier, Rafii Arash, Kimia Kahrizi, Hossein Najmabadi, Hans-Hilger Ropers, and Elizabeth J. Bhoj. 2021. "Discovery of a neuromuscular syndrome caused by biallelic variants in ASCC3." HGG Advances 2 (2):100024. doi: 10.1016/j.xhgg.2021.100024.

Najmabadi, Hossein, Hao Hu, Masoud Garshasbi, Tomasz Zemojtel, Seyedeh Sedigheh Abedini, Wei Chen, Masoumeh Hosseini, Farkhondeh Behjati, Stefan Haas, Payman Jamali, Agnes Zecha, Marzieh Mohseni, Lucia Püttmann, Leyla Nouri Vahid, Corinna Jensen, Lia Abbasi Moheb, Melanie Bienek, Farzaneh Larti, Ines Mueller, Robert Weissmann, Hossein Darvish, Klaus Wrogemann, Valeh Hadavi, Bettina Lipkowitz, Sahar Esmaeeli-Nieh, Dagmar Wieczorek, Roxana Kariminejad, Saghar Ghasemi Firouzabadi, Monika Cohen, Zohreh Fattahi, Imma Rost, Faezeh Mojahedi, Christoph Hertzberg, Atefeh Dehghan, Anna Rajab, Mohammad Javad Soltani Banavandi, Julia Hoffer, Masoumeh Falah, Luciana Musante, Vera Kalscheuer, Reinhard Ullmann, Andreas Walter Kuss, Andreas Tzschach, Kimia Kahrizi, and H. Hilger Ropers. 2011. "Deep sequencing reveals 50 novel genes for recessive cognitive disorders." Nature 478 (7367):57-63. doi: 10.1038/nature10423.
